# Spatio-temporal cluster and distribution of human brucellosis in Shanxi Province of China between 2011 and 2016

**DOI:** 10.1038/s41598-018-34975-7

**Published:** 2018-11-19

**Authors:** Ting Wang, Xiang Wang, Ping Tie, Yongfei Bai, Yuhua Zheng, Changfu Yan, Zhikai Chai, Jing Chen, Huaxiang Rao, Lingjia Zeng, Limin Chen, Lixia Qiu

**Affiliations:** 1 Shanxi Center for Disease Control and Prevention, Taiyuan, 030012 China; 2 China Railway Taiyuan Group Center for Disease Control and Prevention, Disease Control Division, Taiyuan, 030000 China; 3 Shanxi Medical University, School of Public Health, Taiyuan, 030001 China; 4 Qinghai Center for Disease Control and Prevention, Institute for Communicable Disease Control and Prevention, Xining, 810007 China; 5 China Center for Disease Control and Prevention, Beijing, 102206 China

## Abstract

In recent years, the incidence of human brucellosis (HB) in the Shanxi province has ranked to be the top five among the 31 China provinces. HB data in Shanxi province between 2011 and 2016 were collected from the Centers for Disease Control and Prevention. Spatial and temporal distribution of HB was evaluated using spatial autocorrelation analysis and space-time scan analysis. The global Moran’s *I* index ranged from 0.37 to 0.50 between 2011 and 2016 (all *P* < *0*.*05*), and the “high-high” clusters of HB were located at the northern Shanxi, while the “low-low” clusters in the central and southeastern Shanxi. The high-incidence time interval was between March and July with a 2-fold higher risk of HB compared to the other months in the same year. One most likely cluster and three secondary clusters were identified. The radius of the most likely cluster region was 158.03 km containing 10,051 HB cases. Compared to the remaining regions, people dwelling in the most likely region were reported 4.50-fold ascended risk of incident HB. HB cases during the high-risk time interval of each year were more likely to be younger, to be males or to be farmers or herdsman than that during the low-risk time interval. The HB incidence had a significantly high correlation with the number of the cattle or sheep especially in the northern Shanxi. HB in Shanxi showed unique spatio-temporal clustering. Public health concern for HB in Shanxi should give priority to the northern region especially between the late spring and early summer.

## Introduction

Human brucellosis (HB) is the most common zoonotic infection worldwide. Although HB can rarely cause death, it is related with substantial residual disability, physical and psychological dysfunction, and even heavy social and economic burden^1–4^.

The worldwide geographical map of HB has sharply changed over several decades with the ameliorating sanitary conditions, the rapid developing of socioeconomic status, and the increased public health concern. According to the worldwide epidemiology distribution of HB in 2013, low HB incidences were reported from the developed nations^5^. The annual incidence rate was less than two cases per million populations in Canada and the United States of America^6,7^. On contrary, the central Asia and North Africa, especially the regions with advanced animal husbandry but deprived economy, were the new epicenter of HB^8–10^. The highest incidence of HB was reported in Syria and Mongolia, with greater than 500 cases per million in the population, and only two to ten cases in China^2,5^.

However, HB cases have drastically ascended in recent 20 years in China, mostly due to the rapid development of livestock farming and tourism^11,12^. HB cases have increased from 329 in 1993 (0.3 cases per million population) to 47,139 in 2016 (34.4 cases per million population). For the areas in northern China especially Inner Mongolia, Shanxi, Heilongjiang, Jilin and Xinjiang, the HB incidences are extremely high^13^. HB in Shanxi province has always ranked to the top 5 among the 31 Chinese provinces^14,15^. The spatial, temporal, and spatio-temporal distribution of HB in Shanxi undoubtedly plays a critical role in determining public health priority and drawing up the prevention and control strategies for HB in Shanxi^16–18^.

The spatial autocorrelation analysis^19,20^ and space-time scan analysis^7,17,21^ has been widely used to identify the higher-risk areas and periods, as well as to recognize the spatiotemporal variation of epidemics. A spatio-temporal epidemiology for HB needs to be assessed to evaluate the spatial and temporal patterns. To our knowledge, no recent spatial-temporal epidemic of HB has been systematically investigated in Shanxi. Therefore, we combined spatial autocorrelation analysis and space-time scan analysis, and aimed to investigate the spatial and temporal clustering of HB in Shanxi province between 2011 and 2016, and then to identify the HB high-risk regions and high-time^22^.

## Results

### HB incidence in Shanxi province

A total of 38,284 HB cases were reported in Shanxi between 2011 and 2016. The HB incidence showed first an increase and then a decrease in the recent six years, and reached its peak in 2014 (23.53/100,000), and the bottom in 2016 (12.52/100,000), Datong and Shuozhou, located in the most northern Shanxi, showed the two top HB incidence among 11 municipal regions of Shanxi (Table 1). HB incidence rates in males were reported to be 3.67-fold higher than those in females, the unbalanced incidence was also similar with individual 10-years age group (data not shown). The most HB incident cases were the population aged 45 to 60 years and it accounted for 41.91% of all cases; 90.28% of HB cases was found to be farmers or herdsmen (Supplementary Table 1).

**Table 1:** Reported cases and incidence of human brucellosis per 100,000 population of 11 municipal regions in Shanxi China between 2011 and 2016*.

Year	2011**	2012**	2013	2014	2015	2016
	N (1/100,000)	N (1/100,000)	N (1/100,000)	N (1/100,000)	N (1/100,000)	N (1/100,000)
Datong	1,241 (37.40)	1,471 (44.09)	1,795 (53.94)	2,388 (71.36)	1,988 (59.10)	1,186 (34.82)
Shuozhou	929 (54.17)	1,091 (63.14)	1,060 (61.13)	1,073 (61.54)	792 (45.19)	713 (40.46)
Xinzhou	512 (16.69)	561 (18.20)	742 (24.04)	988 (31.85)	789 (25.31)	460 (14.64)
Lvliang	258 (6.92)	324 (8.57)	353 (9.36)	514 (13.55)	462 (12.12)	238 (6.21)
Taiyuan	106 (2.52)	91 (2.17)	124 (2.94)	191 (4.51)	177 (4.16)	91 (2.11)
Yangquan	62 (4.53)	58 (4.22)	65 (4.72)	57 (4.12)	108 (7.77)	109 (7.80)
Jinzhong	817 (25.14)	931 (28.49)	888 (27.06)	996 (30.18)	767 (23.13)	582 (17.45)
Linfen	388 (8.99)	543 (12.48)	635 (14.17)	823 (18.27)	767 (16.94)	553 (12.47)
Changzhi	246 (7.38)	345 (10.28)	367 (10.96)	410 (12.18)	309 (9.14)	232 (6.78)
Jincheng	99 (4.34)	113 (4.93)	105 (4.57)	107 (4.65)	82 (3.55)	66 (2.85)
Yuncheng	474 (9.23)	601 (11.62)	761 (14.66)	993 (19.02)	756 (14.41)	357 (6.77)
Total	5,135 (14.38)	6,130 (17.06)	6,895 (19.10)	8,540 (23.53)	6,997 (19.18)	4,587 (12.52)

*All regions were ranked from the northern Shanxi to southern Shanxi according to their geographic location.

**All 11 municipal regions were shown in the Fig. 5; There were three cases in 2011 and one case in 2012 lack of detailed administrative regions.

**Figure 1:**
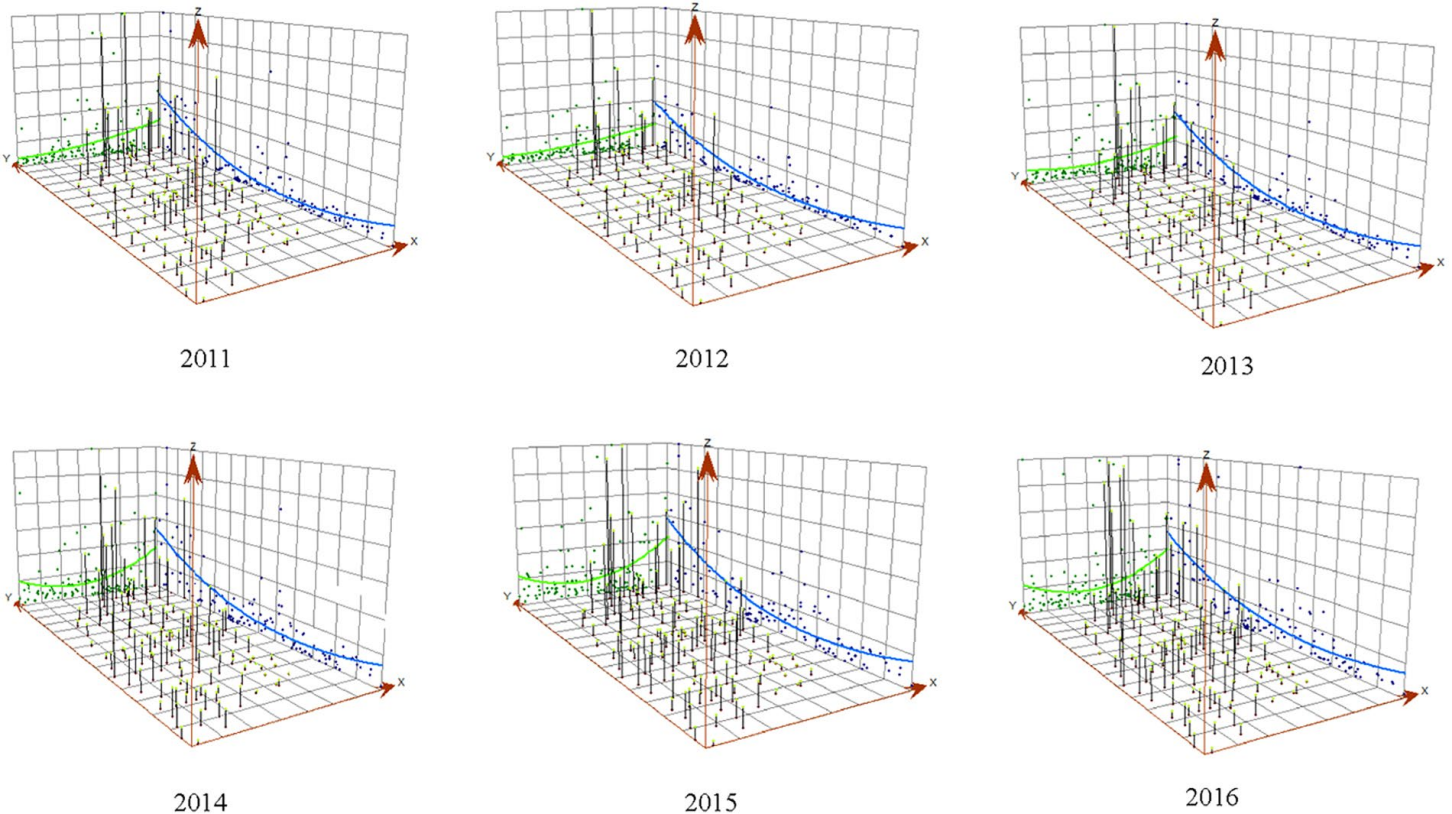
Three-dimensional trend of the annual incidence rate of human brucellosis in Shanxi, China between 2011 and 2016*. X- and Y-axis represent the longitude (from west to east) and latitude (from south to north) of the geometric center of Shanxi province in China, respectively, and Z-axis represents the HB incidence, that is, one point (X, Y, Z) indicates some specific study region, and higher Z value means higher HB incidence.

The overall 3-dimensional trend for each year indicated an even distribution of HB in Shanxi every year (Fig. 1), HB incidences in the northern region were higher than the southern region. Similarly, the eastern region had higher HB incidence than the western region. HB incidences for most of central and southern regions were less than 30 per 100,000 people, while ≥30/100,000 incidences were more frequently observed in the northern region, mainly in the Youyu, Xinrong, Zuoyun, Tianzhen and Yonghe counties or districts.

### Distribution of four different clusters

**Figure 2:**
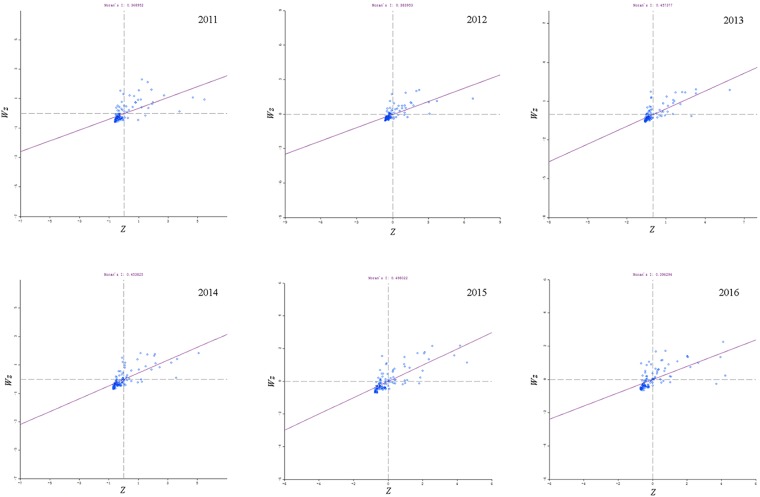
Global Moran’s I scatter plot of human brucellosis incidence in 119 counties (districts) of Shanxi, China between 2011 and 2016*, *The horizontal axis of the Moran scatter plot, is the observed and normalized z-score $({Z}_{i}=\frac{{X}_{i}-X}{s})$ for specific county or district, and the vertical axis is the weighted sum of observed and normalized z-score for the neighboring counties or districts $({{\rm{Wz}}}_{i}=\sum _{i=1}^{n}{w}_{ij}{z}_{j})$. The individual dots display the specific 119 counties or districts. The first to fourth quadrants of the Moran scatter plot correspond to the high-high, low-high, low-low and high-low correlations for local Moran’s *I*.

**Figure 3:**
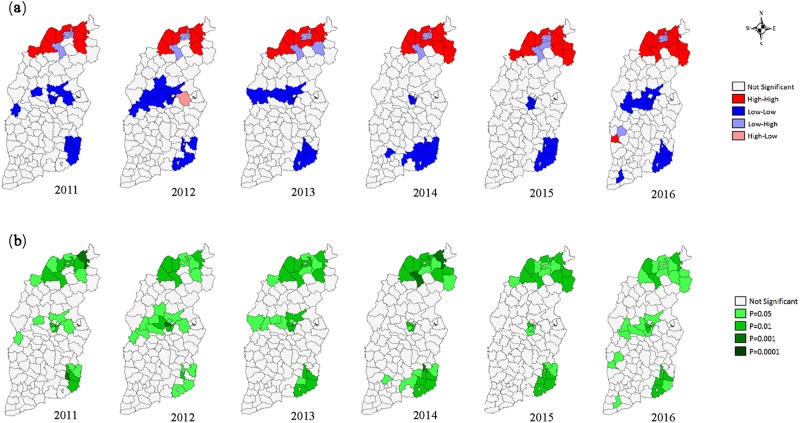
Local Moran’s *I* diagram of human brucellosis incidence among 119 counties or districts of Shanxi, China between 2011 and 2016*. * (**a**) Local Moran’s I cluster map. (**b**) Local Moran’s I significance map.

The HB incidences between 2011 and 2016 in individual counties or districts showed significant spatial autocorrelation and spatial cluster, their global Moran’s *I* index ranged from 0.369 to 0.498 (*P* < 0.05) (Supplementary Table 2). Most of the cluster dots in the Moran scatter plot were in the first and third quadrants, with the most number of dots observed in the third quadrant and the least in the fourth quadrant (Fig. 2). The four different types of local spatial autocorrelation clusters emerged concurrently and were visualized by local Moran’s *I* cluster map (Fig. 3a) and significance map (Fig. 3b).

Combining the spatial autocorrelation and the Moran scatter plot, we found that the main clusters were the low-low ones, followed by the high-high clusters, and the high-low clusters were the least frequent. The low-low clusters focused on three cities (Taiyuan, Changzhi and Jincheng) located in the central Shanxi; the high-high clusters focused on four counties (Gaoyang, Datong, Hunyuan and Zuoyun) of the Datong city, and the two counties (Youyu and Pinglu) of Shuozhou city adjacent to the Datong city, which were in the northern regions; Nanjiao district of the Datong city and Shanyin county of the Shuozhou city were the low-high clusters; Shouyang county of the Jinzhong city was the high-low cluster.

### Spatio-temporal distribution

The purely temporal scan analysis between 2011 and 2016 showed that the high-incidence seasons of HB was the time interval between each late spring and each early summer. The peak months in 2011, 2015, and 2016 was observed to be April to July, whereas from 2012 to 2014, it was March to June. Compared to the other seasons, the high-incidence seasons also showed 1.80- to 2.10-fold increased risk for HB (Table 2).

**Table 2:** Purely temporal scan analysis of human brucellosis in Shanxi, China between 2011 and 2016.

Year	Cluster time frame	Observed cases	Expected cases	*LLR*	*P*	*RR*
2011	2011/4/1 to 2011/7/31	2,636	1,715	349.19	<*0*.*001*	2.10
2012	2012/3/1 to 2012/6/30	3,026	2,041	336.67	<*0*.*001*	1.95
2013	2013/3/1 to 2013/6/30	3,350	2,305	336.93	<*0*.*001*	1.88
2014	2014/3/1 to 2014/6/30	4,241	2,855	476.76	<*0*.*001*	1.96
2015	2015/4/1 to 2015/7/31	3,546	2,339	440.66	<*0*.*001*	2.05
2016	2016/4/1 to 2016/7/31	2,171	1,529	191.77	<*0*.*001*	1.80

**Figure 4:**
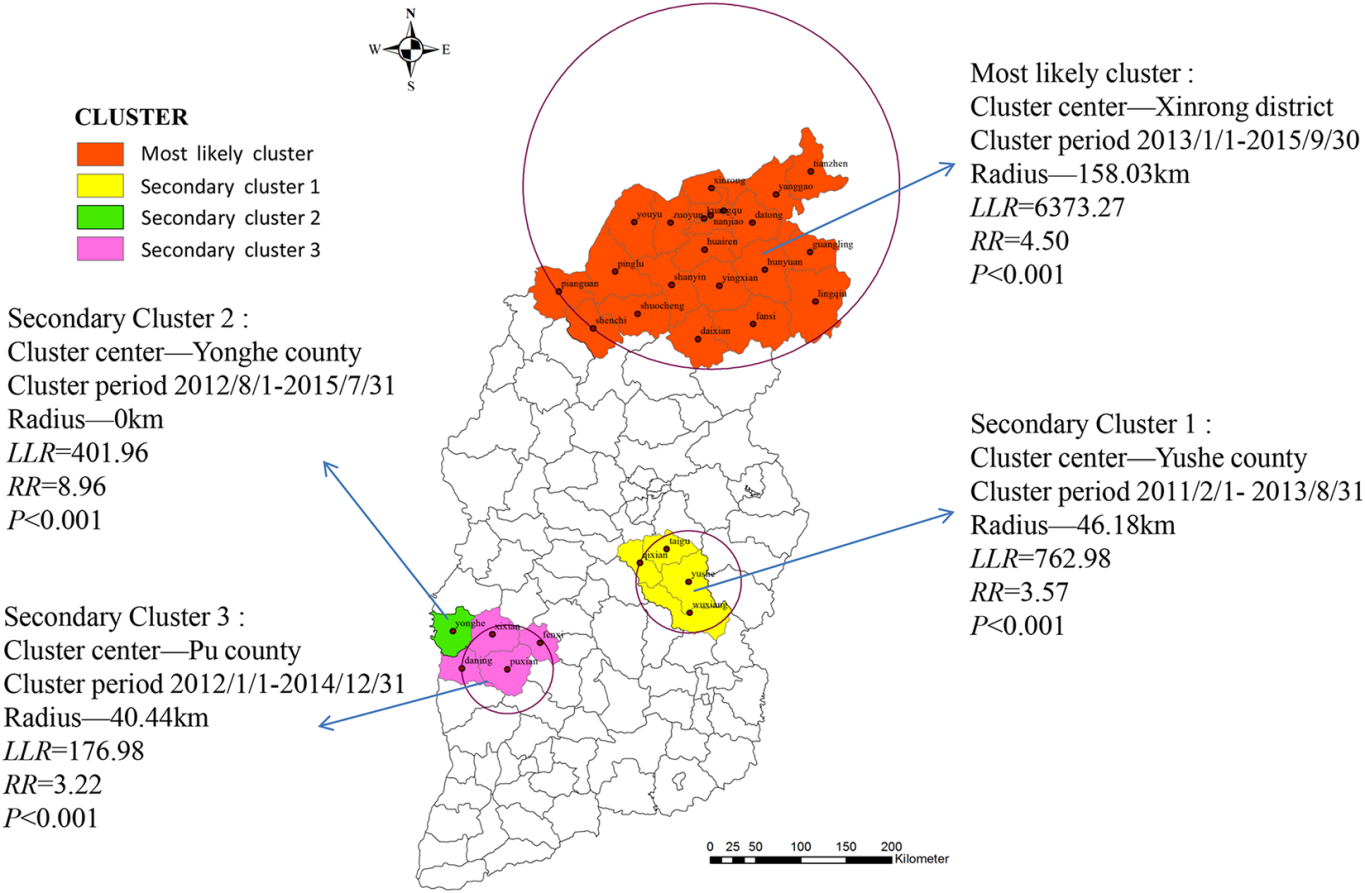
Spatio-temporal clusters of human brucellosis among 119 counties or districts in Shanxi, China between 2011 and 2016.

The spatio-temporal scan analysis to HB incidence of the 119 counties or districts showed that four significant spatio-temporal clusters were found, including one most likely cluster and three secondary likely clusters (Fig. 4). The most likely cluster region was centered at Xinrong district in the Datong city, the radius of the cluster region was 158.03 km, which covered 21 counties or districts (11 in Datong city, 6 in Shuozhou city, and 4 in Xinzhou city), and 10,051 HB cases. This most likely cluster region also reported the peak incidence between January 1, 2013 and September 30, 2015. Comparing the rest regions, this most likely cluster was reported to have 4.50-fold increased risk for HB incidences, the three secondary likely clusters also were observed to have between 3.22- and 8.96-fold elevated risks for HB.

### Potential risk factors for current spatio-temporal clusters

HB cases during the high-risk (HR) time interval were more likely to be younger, to be males or to be farmers or herdsman than that during the low-risk time interval from 2011 to 2016 in Shanxi (Table 3). Multivariate logistic regression also showed that age, sex and current occupation were independent risk factors of high HB incidence of the HR time interval; the young cases aged 30–45 years vs. the cases aged more than 60 years had a 1.22-fold increased HB risk among the March to July between 2010 and 2016 (95%CI, 1.15–1.30); the male cases and the farmers or herdsman vs. the unemployment or retirees had a 1.12-fold and 1.17-fold) increased risk of HB in the HR time interval, respectively.

**Table 3:** Difference of age, gender and current occupation between high-risk time interval and low-risk time interval among each year from 2011 to 2016 in Shanxi province.

	2011		2012		2013		2014		2015		2016	
	HR time interval	LR time interval	HR time interval	LR time interval	HR time interval	LR time interval	HR time interval	LR time interval	HR time interval	LR time interval	HR time interval	LR time interval
**Age (years)**												
0–15	76 (2.4)	31 (1.6)	78 (2.1)	26 (1.1)	91 (2.3)	51 (1.8)	117 (2.3)	52 (1.5)	92 (2.2)	59 (2.2)	58 (2.2)	27 (1.4)
16–30	325 (10.3)	111 (5.6)	323 (8.9)	160 (6.4)	401 (10.0)	192 (6.6)	509 (10.1)	199 (5.7)	399 (9.3)	151 (5.6)	269 (10.3)	139 (7.1)
31–45	878 (27.8)	413 (20.9)	1,005 (27.6)	503 (20.2)	1,018 (25.5)	531 (18.3)	1,261 (24.9)	712 (20.5)	966 (22.5)	433 (16.0)	560 (21.3)	329 (16.8)
46–60	1,340 (42.4)	826 (41.8)	1,550 (42.5)	1,003 (40.4)	1,675 (41.9)	1,232(42.6)	2,140 (42.3)	1,380 (39.7)	1,844 (43.0)	1,131 (41.8)	1,138 (43.4)	786 (40.0)
61–75	524 (16.6)	501 (25.4)	659 (18.1)	675 (27.2)	777 (19.4)	749 (25.9)	965 (19.1)	1,023 (29.4)	928 (21.6)	830 (30.6)	559 (21.3)	610 (31.1)
>75	16 (0.5)	94 (4.8)	29 (0.8)	119 (4.8)	38 (1.0)	140 (4.8)	73 (1.4)	109 (3.1)	59 (1.4)	105 (3.9)	40 (1.5)	72 (3.7)
*P*	***<0***.***001***		***<0***.***001***		***<0***.***001***		***<0***.***001***		***<0***.***001***		***<0***.***001***	
**Gender**												
Male	2,611 (82.7)	1,527 (77.3)	2,979 (81.8)	1,927 (77.5)	3,175 (79.4)	2,204 (76.1)	4,048 (79.9)	2,650 (763)	3,351 (78.2)	2,043 (75.4)	2,067 (78.7)	1,503 (76.6)
Female	548 (17.3)	449 (22.7)	665 (18.2)	559 (22.5)	825 (20.6)	691 (23.9)	1017 (20.1)	825 (23.7)	937 (21.8)	611 (24.6)	557 (21.3)	460 (23.4)
*P*	***<0***.***001***		***<0***.***001***		***<0***.***001***		***<0***.***001***		***0***.***255***		***0***.***075***	
**Occupation**												
Farmer	2,640 (83.6)	1,607 (81.3)	3,130 (85.9)	2,048 (82.4)	3,320 (83.0)	2,318 (80.1)	4,070 (80.4)	2,719 (78.2)	3,554 (82.9)	2,248 (83.0)	2,206 (84.1)	1,692 (86.2)
Herdsman	258 (8.2)	143 (7.2)	274 (7.5)	197 (7.9)	296 (7.4)	218 (7.5)	520 (10.3)	369 (10.6)	329 (7.7)	188 (6.9)	142 (5.4)	77 (3.9)
Unemployed and retirees	19 (0.6)	95 (4.8)	14 (0.4)	90 (3.6)	28 (0.7)	66 (2.3)	22 (0.4)	88 (2.5)	34 (0.8)	65 (2.4)	25 (1.0)	57 (2.9)
Student	73 (2.3)	33 (1.7)	49 (1.3)	31 (1.3)	63 (1.6)	52 (1.8)	81(1.6)	53 (1.5)	54 (1.3)	32 (1.2)	49 (1.9)	24 (1.2)
Worker	28(0.9)	24 (1.2)	35 (1.0)	27 (1.1)	47 (1.2)	17 (0.6)	50 (1.0)	26 (0.8)	38 (0.9)	27 (1.0)	78 (3.0)	36 (1.8)
Others	141 (4.5)	74 (3.7)	142 (3.9)	93 (3.7)	246 (6.2)	224 (7.7)	322 (6.4)	220 (6.3)	279 (6.5)	149 (5.5)	124 (4.7)	77 (3.9)
*P*	***<0***.***001***		***<0***.***001***		***<0***.***001***		***<0***.***001***		***<0***.***001***		***<0***.***001***	

Abbreviations: HR: high-risk; LR: low-risk.

**Figure 5:**
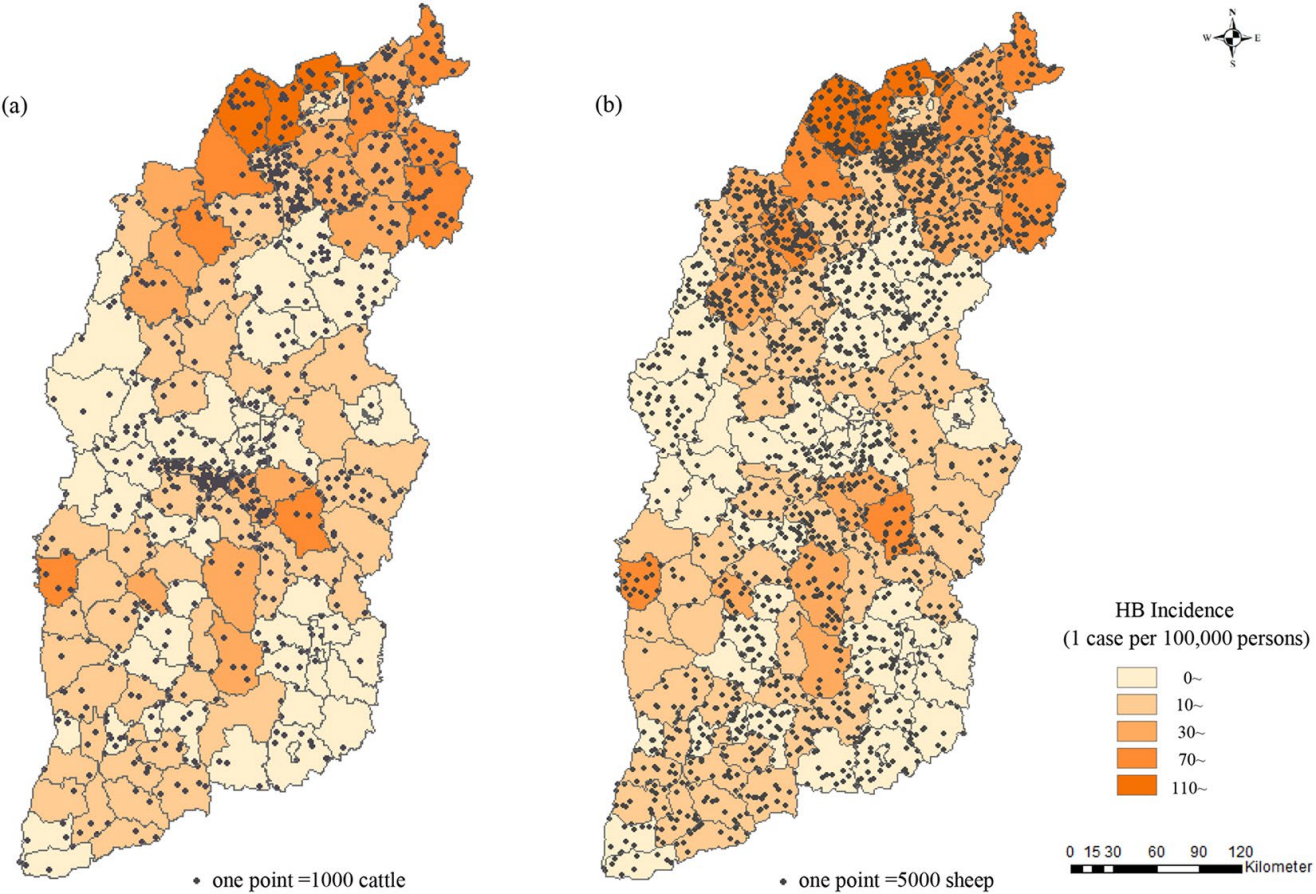
The distribution of average HB incidence and the average number of sheep or cattle of 119 counties or districts of Shanxi province across six years from 2011 to 2016*. *(**a**,**b**) indicated the distribution of average HB incidence and the average number of cattle or sheep, one solid circle represented 1,000 cattle (**a**) or 5,000 sheep (**b**).

The HB incidence had a significantly high correlation with the number of the cattle or sheep, their correlation coefficients were about 0.5 or more (*all P* < *0*.*05*), it also showed a relative low correlation with the number of pigs (Supplementary Table 3). We also visualized the distribution of average HB incidence and the average number of sheep or cattle across six years of 119 counties or districts (Fig. 5), it showed that most of areas had a greater number of sheep than the number of cattle, this situation was more obvious in Datong and Shuozhou counties, which located in the most northern Shanxi and also had the two top HB incidence. Multivariate linear regression also showed that the number of sheep and the northern Shanxi were significantly independent factors for the high incidence of HB (*P* = *0*.*002; P* = *0*.*005*) after adjusting for the number of sheep, cattle and pigs, and the geographic position.

## Discussion

Our study investigated the spatial and temporal distribution of HB incidences in the Shanxi province, based on the population-based monitoring data between 2011 and 2016. HB in Shanxi showed unique spatio-temporal clustering. The “high-high” clusters of HB were located at the northern Shanxi, and the “low-low” clusters at the central and southeastern Shanxi, with the high-incidence time interval focused between March and July.

The spatial autocorrelation analysis showed significant spatial clustering of HB incidences in Shanxi province between 2011 and 2016 but varied by different counties or districts^23^. This spatial distribution of HB may be attributed to the different reasons. Among these, the development and spreading of stock farming should be considered, as it plays a critical and direct role in HB incidence in Shanxi province. HB is a natural-focal disease^15^, and the “high-high” regions shares similar natural and social environments. The “high-high” clusters in Shanxi were adjacent to Inner Mongolia, a region with the highest HB incidences across China in recent years^14^. Same as Inner Mongolia, most of these identified regions were covered with large proportion of grasslands and animal husbandry was their main industry. With the expansion and industrialization of livestock husbandry, large numbers of livestock farmers were required and engaged in manufacturing of cow and sheep-related products. This can also possibly result in increasing the risk of HB epidemic across these regions^12,24^. The fact that the “high-high” cluster regions have expanded from 8 counties in 2011 to 12 counties in 2016 is in accordance to our hypothesis (data not shown). Counties like Huairen from Shouzhou city in 2013, Guangling and Lingqiu from Datong city in 2014, as well as Daning from Linfen city in 2016, gradually became the part of the “high-high” clusters. A review from Turkey reported that the highest incidence of HB was found in abattoir workers, in addition, veterinarians, veterinary assistants and slaughterhouse workers had 4.8% higher chance of being contracted HB than the nonprofessional population^25^.

Another potential reason of spatial distribution of HB is the relative economic disadvantage of “high-high” counties or districts. The general populations from these areas have low awareness and poor knowledge for HB prevention and control^2,26,27^. A bad habit of eating raw lamb and drinking raw goat milk can also greatly increase the risk of HB^28^. The residents with poor sanitary conditions can be easily exposed to Brucella-contaminated food and water sources. In addition, poor access to immediate treatment after infection worsens the HB incidence. The increase in the risk of HB infection through raw milk is in accordance to another study from Greece published in 2016^29^. One review paper enumerated the important effect of economy on the global epidemic of HB in 2006^2^. On contrary, the “low-low” clusters in Shanxi that includes Taiyuan, Changzhi and Jincheng cities have economic superiority, standardized industrial manufacturing for cow or sheep related products, good sanitary habits and awareness for HB.

The purely temporal scan analysis revealed the peak time of HB incidence in Shanxi, mainly focusing on the time interval from March to July. The period between March and July was the peak delivery time of domestic livestock such as cows and lambs, and farmers are highly involved in delivering the livestock as well as handling the newly-born animals, therefore are more prone to be exposed to HB^15,24,28^. Then the acute HB appeared in a short time because of the incubation of only one to three weeks for HB. Our study also found that HB cases during the high-risk time interval were more likely to be younger, to be males or to be farmers or herdsman because the male was more prone to be engaged in the animal husbandry, which can partly explain the time distribution of HB in Shanxi. The seasonal factors of HB in Shanxi are in accordance to the peak time reported in Iran^30^ between 2011 and 2014. There was a similar report in the countries with temperate or cold climates, a markedly increased variation in the incidence of brucellosis appeared in the spring and summer due to the high exposure of those attending the animals and consuming their milk^15,30^. This situation was further strengthened for ovine/caprine brucellosis and for bovine brucellosis, possibly because of the longer lactation period in cattle. It is important to note that the main livestock for the northern pastoral areas was just the ovine/caprine^28^.

The space-time scan analysis also identified one most likely cluster and three secondary likely clusters. The geometric center for the most likely cluster region was Xinrong district of Datong city, the cluster covered 21 counties from Datong, Shuozhou and Xinzhou cities. This cluster area had the highest HB incidence, large cluster radius and 4.5-fold increased relative risk of HB compared with the remaining areas of Shanxi, and these results were in accordance with the previous spatial autocorrelation analysis. This huge endemic area can owe much to its stock farming as mainstay industry for the northern Shanxi^15^, which could be partly supported by our results that the HB incidence of the northern Shanxi had relative high correlation with the number of sheep or cattle, and the correlation coefficient with the number of sheep was bigger than with the number of cattle, and previous studies also confirmed that the sheep or cattle had relative high ability to cause HB. Another reason could be the frequent exchange of the infected products from the pastoral region to its adjacent areas^3^. With the advancement of animal husbandry, frequent circulation of animal products and insufficient quarantine, farmers would sell the infected animals at a low price to minimize economic losses, which may have resulted in the spread of epidemic situation into more regions^5,8^. Although the three secondary likely cluster areas had relatively smaller cluster radius, they still contained the higher relative risk of HB. Disease prevention should still be reinforced to decrease the HB infection, all four clusters should become the newest focus of public health for preventing and controlling HB in Shanxi.

Our study has two main strengths. Firstly, the data in our study was collected depending on the large-scale and population-based monitoring system of Shanxi province for continuously six years, which provides solid support for our detailed evaluation. Secondly, the spatial and temporal patterns in Shanxi were synthetically investigated using the geographic information system and the spatial-temporal scan. This conceals the weakness of the traditional statistical analysis method and can efficiently identify higher-risk areas and periods and recognize the spatiotemporal variation of epidemics HB.

However, there are few shortcomings in our study. Firstly, the HB incidence was underestimated to some extent, because our data was passively collected by depending on a monitoring system, while the surveillance data quality was influenced by some comprehensive factors, such as the capacity of the local health workers, the availability of laboratory diagnostics, the awareness of potential cases to visit doctors and so on. Given Shanxi is a relative high-risk area for HB, the health workers have more experience for HB detection. However, under-reporting is a world-wide problem in any surveillance system especially for HB incident cases, the HB data from the surveillance system for relative large-scale population has still better capacity of providing the effective information for public health prevention. Secondly, similar with several previous studies^6,30–32^, we only could collect few the demographic characteristics such as age, sex and occupation, the data of occupation had some misclassification between herdsman and farmer, other potential risk factors of the population couldn’t be collected due to the phrasing of the relevant reporting form item. However, in order to support the fact that the relative high HB incidence was ascribed to the development of agriculture and husbandry^31,33^, therefore, we evaluated that correlation strength between the number of cattle, pigs or sheep and the HB incidence using the data from Shanxi Statistical Information Network, the results that the total number of the cattle or sheep had a relative high correlation with HB incidence, could explain the current spatio-temporal distribution of HB of Shanxi province to some extent. Occupation was also confirmed to be another important risk factor for HB, consistent results with other similar studies was shown that the herdsman had more HB incidence than other kinds of occupation. Thirdly, we couldn’t collect the information of main subtype of the HB cases infected from sheep, cattle, pigs or dogs. However, our correlation analysis showed the total number of sheep had higher correlation with HB incidence than the total number of cattle, and the total number of pigs had relative low correlation with HB incidence, these results were also similar with previous studies^1,9,34^; while the correlation with the total number of dogs couldn’t be evaluated due to a lack of the report of the total number of dogs^35^.

Our study provides the most scientific evidence into the future allocation of health resources and reshaping of the prevention and control strategies of HB epidemic in the Shanxi province. The public health of the population from Shanxi should be given priority; especially for the northern Datong and Shuozhou cities in every late spring and early summer, effective measures and control strategies should be executed. Furthermore, training and education should be reinforced and launched in the high-incidence areas^36,37^.

## Methods

### Data collection

#### HB cases

Our study was an observational study based on the disease monitoring system of Shanxi Province, which locates in the northern China and is a typical loess-covered mountainous plateau (its geographic map is shown in the Supplemental Fig. 1).The HB cases and their related information between Jan 1, 2011 and Dec 31, 2016, were obtained depending on the China Information System for Disease Control and Prevention (CISDCP, http://1.202.129.170/UVSSERVER2.0). All eligible HB cases currently resided in the Shanxi province, were diagnosed between Jan 1, 2011 and Dec 31, 2016 and were ascertained by clinical indicators and laboratory tests, and the following cases were excluded: foreigners, China Hong Kong, Macao or Taiwan cases; double reported cases; misclassified cases.

#### Geography information

Shanxi Province electronic map file (1:1 000 000), including latitude and longitude data of individual 119 counties or districts, was downloaded from the National Earth System Science Data Sharing Platform (http://www.geodata.cn).

#### Baseline characteristic and risk factors

The demographic information (sex, age and occupation) for all HB cases were collected by monitoring system; the number of cattle, pigs and sheep of each end of year in Shanxi Province from 2011 to 2016 were collected from Shanxi Statistical Information Network (http://www.stats-sx.gov.cn).

### Statistical analysis

The HB incidence in Shanxi province in every year from 2011 to 2016 for 11 districts of Shanxi Province was summarized using the proportion. We defined each county (district) as the cluster unit, all cases were classified into corresponding 119 counties or districts according to their reported currently dwelling address and postal area code. The spatial distribution and incidence trend of HB were evaluated and visualized by the three-dimensional (3D) trend analysis using ArcGIS10.2.2 software (ESRI, Redlands, CA, USA). The X- and Y-axis represent the geometric center of specific study region, and Z-axis represents the HB incidence^38^.

Spatial correlation strength in Shanxi was evaluated by spatial autocorrelation analysis using OpenGeoDa^39,40^ (GeoDa Center for GeospatialAnalysis and Computation, Arizona State University, AZ, USA). Global Moran’s *I* index value was used to describe the global autocorrelation among all 119 counties or districts^41^. Local Moran’s *I* index was used to evaluate the correlation between individual target region and the rest of the neighboring regions^42,43^. Transformed Z test is used to test the Moran’s *I* index^16,41,44^. Global and local Moran’s *I* were visualized by the Moran scatter plot, the slope of the line fitted by the scatter plot equaled to the global Moran’s *I* index value. The first to fourth quadrants of the Moran scatter plot correspond to the high-high, low-high, low-low and high-low correlations for local Moran’s *I*^23,38,45^. The four different types of local correlation and significance of corresponding Moran’s *I* index were visualized using local Moran’s *I* significance map and by cluster map in various color.

Cluster place, time and clustering strength of HB clustering, was investigated by space-time scan analysis using the SaTScan9.4.1 and being visualized by ArcGIS10.2.2. The average HB incidence of Shanxi province between 2011 and 2016 equaled to total number of incident cases divided by total population, and the average number of cattle, pigs or sheep between 2011 and 2016 equaled to the total number of cattle, pigs or sheep divided by six. We had the hypothesis that HB incidence is subject to Poisson distribution, and scanning window for each county or district could cover 50% of the overall population, the least cluster time interval was 6 months^9,38^, then the most likely cluster, the secondary likely clusters, or secondary likely cluster 3 and so on among 119 regions in Shanxi province were determined by log-likelihood ratio (LLR)^7,21,46^.

*P* value was calculated by Monte Carlo randomization with sampling times of 999. The relative risk (RR) was calculated to evaluate HB risk of the high-risk (HR) time interval by comparing HB incidence of the cluster time interval with that of the rest time of the same year (low-risk (LR) time interval), and the cluster strength though comparing the incidence of target radius with that of the rest regions^47,48^.

The distribution difference of age, sex and current occupation was compared across LR time interval and high-risk time interval between 2011 and 2016, the unconditional logistic regression was also used to investigate the potential cofounders of the high HB incidence in the HR time interval by adjusting for age (0–15, 16–29, 31–45, 46–60, or ≥61 years), sex (male or female) and current occupation (farmer, herdsman, unemployment and retirees, student, worker, or others). To evaluate the potential effect of the number of cattle, pigs and sheep on the HB incidence between 2011 to 2016, the Pearson correction coefficients were calculated and tested using t-test, the multivariate linear regression was also conducted to further determine their effect on the HB incidence after adjusting for the number of sheep, cattle and pigs, and the geographic position (northern counties, middle counties and southern counties) treating one county as the analysis unit. These rest statistical analysis was conducted using the SPSS 22.0 (Statistical Product and Service Solutions), all significance level was 0.05 at two-tailed.

### Ethics and consent

Our study data were collected according to the China Information System for Disease Control and Prevention (CISDCP, http://1.202.129.170/UVSSERVER2. 0). Our study was approved by the ethics review committee of Shanxi Center for Disease Control and Prevention. All methods were performed in accordance with the relevant guidelines and regulations.

## Electronic supplementary material

Supplemental information
