# Supplementary material for: Spatio-temporal cluster and distribution of human brucellosis in Shanxi Province of China between 2011 and 2016
Source: Sci Rep. 2018 Nov 19;8:16977. doi: 10.1038/s41598-018-34975-7 (PMC6242928; doi:10.1038/s41598-018-34975-7)

**Spatio-temporal cluster and distribution of human brucellosis in Shanxi Province of China between 2011 and 2016**

Ting Wang^1,+^, Xiang Wang^2,3,+^, Ping Tie^1,+^, Yongfei Bai^1^, Yuhua Zheng^1^, Changfu Yan^1^, Zhikai Chai ^1^, Jing Chen^1^, Huaxiang Rao^4,+^, Lingjia Zeng^5^, Limin Chen^1,^*, Lixia Qiu^3,^*

1 Shanxi Center for Disease Control and Prevention, Taiyuan, 030012, China

2 China Railway Taiyuan Group Center for Disease Control and Prevention, Disease Control Division, Taiyuan, 030000, China

3 Shanxi Medical University, School of Public Health, Taiyuan, 030001, China

4 Qinghai Center for Disease Control and Prevention, Institute for Communicable Disease Control and Prevention, Xining, 810007, China

5 China Center for Disease Control and Prevention, Beijing, 102206, China

+These authors contributed equally to this work

*Correspondence and requests for materials should be addressed to L.M.C. (e-mail: sxchenlimin@163.com) or L.X.Q. (e-mail: qlx_1126@163.com)

**Supplementary Table 1. The demographic characteristics of human brucellosis cases in Shanxi, China between 2011 and 2016**

| **Variables** | **2011** | **2012** | **2013** | **2014** | **2015** | **2016** | **Total** |
| --- | --- | --- | --- | --- | --- | --- | --- |
| **Age (years)** |  |  |  |  |  |  |  |
| 0-15 | 107(2.08) | 104(1.70) | 142(2.06) | 169(1.98) | 151(2.16) | 85(1.85) | 758(1.98) |
| 15-30 | 436(8.49) | 483(7.88) | 593(8.60) | 708(8.29) | 550(7.86) | 408(8.90) | 3,178(8.30) |
| 30-45 | 1,291(25.14) | 1,508(24.60) | 1,549(22.47) | 1,973(23.1) | 1,399(19.99) | 889(19.38) | 8,609(22.49) |
| 45-60 | 2,166(42.18) | 2,553(41.65) | 2,907(42.16) | 3,520(41.22) | 2,975(42.52) | 1,924(41.94) | 16,045(41.91) |
| 60-75 | 1,025(19.96) | 1,334(21.76) | 1,526(22.13) | 1,988(23.28) | 1,758(25.13) | 1,169(25.49) | 8,800(22.99) |
| >75 | 110(2.14) | 148(2.41) | 178(2.58) | 182(2.13) | 164(2.34) | 112(2.44) | 894(2.34) |
| **Gender** |  |  |  |  |  |  |  |
| Male | 4,138(80.58) | 4,906(80.03) | 5,379(78.01) | 6,698(78.43) | 5,394(77.09) | 3,570(77.83) | 30,085(78.58) |
| Female | 997(19.42) | 1,224(19.97) | 1,516(21.99) | 1,842(21.57) | 1,603(22.91) | 1,017(22.17) | 8,199(21.42) |
| **Occupation at diagnosis** | | |  |  |  |  |  |
| Farmer | 4,247(82.71) | 5,178(84.47) | 5,638(81.77) | 6,789(79.50) | 5,802(82.92) | 3,898(84.98) | 31,552(82.42) |
| Herdsman | 401(7.81) | 471(7.68) | 514(7.45) | 889(10.41) | 517(7.39) | 219(4.77) | 3,011(7.86) |
| Unemployed and retirees | 114(2.22) | 104(1.70) | 94(1.36) | 110(1.29) | 99(1.41) | 82(1.79) | 603(1.58) |
| Student | 106(2.06) | 80(1.31) | 115(1.67) | 134(1.57) | 86(1.23) | 73(1.59) | 594(1.55) |
| Worker | 52(1.01) | 62(1.01) | 64(0.93) | 76(0.89) | 65(0.93) | 114(2.49) | 433(1.13) |
| Others | 215(4.19) | 135(2.20) | 470(6.82) | 542(6.35) | 428(6.12) | 201(4.38) | 2,091(5.46) |

* Others include teacher, medical personnel, attendant and so on.

**Supplementary Table 2. Global spatial autocorrelation analysis of the incidence human brucellosis in Shanxi, China between 2011and 2016**

| **Year** | **Moran’s *I*** | ***V*(I)** | ***Z*-score** | ***P*** |
| --- | --- | --- | --- | --- |
| **2011** | 0.3690 | 0.0521 | 7.2457 | *<0.001* |
| **2012** | 0.3840 | 0.0526 | 7.4620 | *<0.001* |
| **2013** | 0.4574 | 0.0578 | 8.0606 | *<0.001* |
| **2014** | 0.4538 | 0.0572 | 8.0822 | *<0.001* |
| **2015** | 0.4980 | 0.0585 | 8.6581 | *<0.001* |
| **2016** | 0.3963 | 0.0580 | 6.9565 | *<0.001* |

**Supplementary Table 3. Spearmen correlation between the number of cattle, pigs or sheep, or the agriculture or husbandry output and the HB incidence of 119 counties or districts in Shanxi of China between 2011 and 2016***

|  | **2011** | **2012** | **2013** | **2014** | **2015** | **2016** |
| --- | --- | --- | --- | --- | --- | --- |
|  | **r (*P*)** | **r (*P*)** | **r (*P*)** | **r (*P*)** | **r (*P*)** | **r (*P*)** |
| **Cattle** | 0.491  (***＜0.001***) | 0.419  (***＜0.001***) | 0.451  (***＜0.001***) | 0.501  (***＜0.001***) | 0.475  (***＜0.001***) | 0.479  (***＜0.001***) |
| **Sheep** | 0.656  *(****＜0.001****)* | 0.534  *(****＜0.001****)* | 0.507  *(****＜0.001****)* | 0.542  *(****＜0.001****)* | 0.518  *(****＜0.001****)* | 0.568  *(****＜0.001****)* |
| **Pig** | 0.111  *(0.231)* | -0.001  *(0.989)* | -0.102  *(0.279)* | -0.139  *(0.131)* | -0.123  *(0.182)* | -0.065  *(0.480)* |

**Supplementary Figure 1. The geographical location and administrative divisions as the municipal and township level of Shanxi, China**


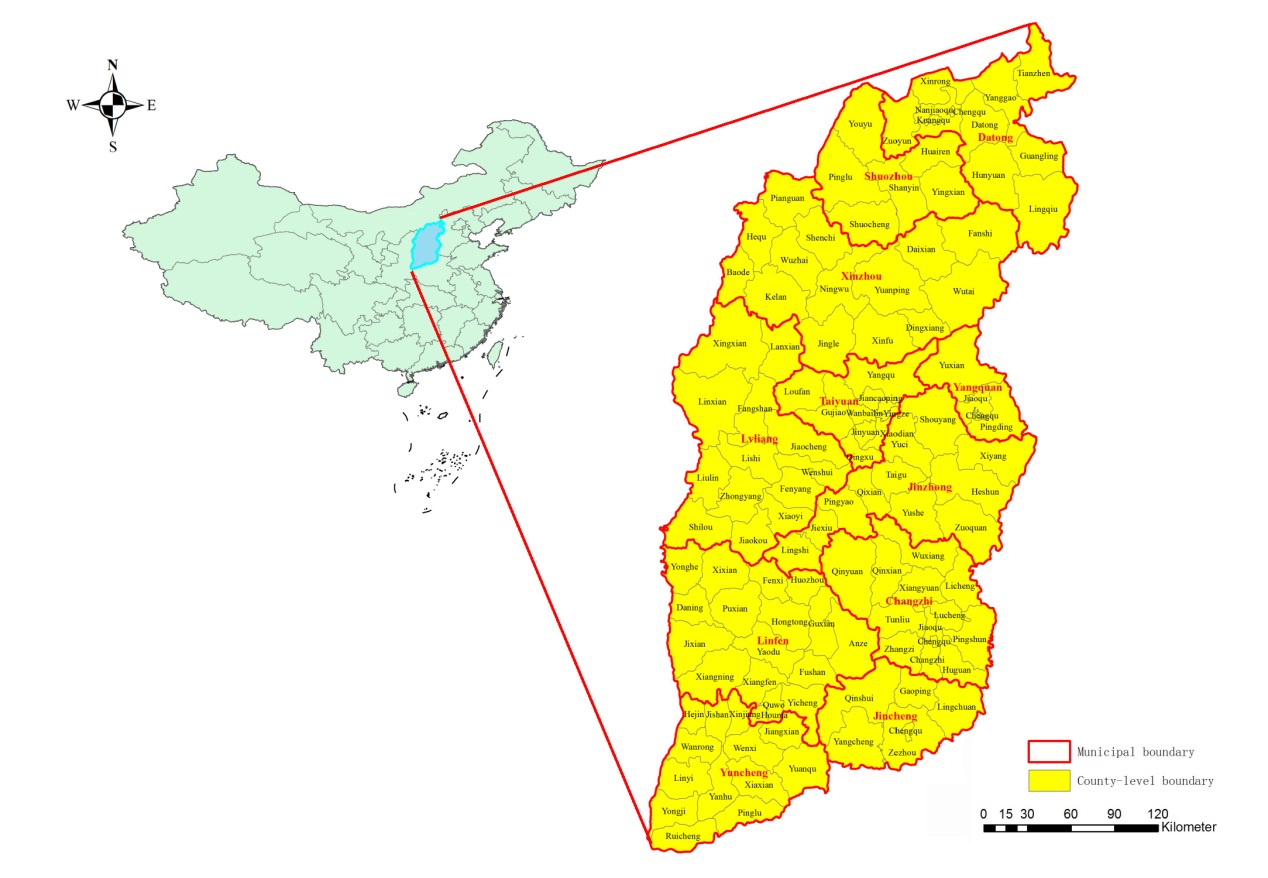

Supplement: Supplementary file 1 — Supplemental information [file 41598_2018_34975_MOESM1_ESM.docx]
